# Facile synthesis of nitrophenyl 2-acetamido-2-deoxy-α-D-mannopyranosides from ManNAc-oxazoline

**DOI:** 10.3762/bjoc.8.48

**Published:** 2012-03-20

**Authors:** Karel Křenek, Petr Šimon, Lenka Weignerová, Barbora Fliedrová, Marek Kuzma, Vladimír Křen

**Affiliations:** 1 Institute of Microbiology, Academy of Sciences of the Czech Republic, Vídeňská 1083, CZ – 142 20, Prague, Czech Republic; 2 Department of Biochemistry, Charles University in Prague, Hlavova 8, CZ – 128 40, Prague, Czech Republic

**Keywords:** α-ManNAc, glycosidase, glycosylation, nitrophenyl, oxazoline

## Abstract

The synthetic procedures for a large-scale preparation of *o*- and *p*-nitrophenyl 2-acetamido-2-deoxy-α-D-mannopyranoside are described. The synthetic pathway employs the glycosylation of phenol with ManNAc oxazoline, followed by nitration of the aromatic moiety yielding a separable mixture of the *o*- and *p*-nitrophenyl derivative in a 2:3 ratio.

## Introduction

Hexosamines are fundamental structural elements and precursors of the peptidoglycan and membrane lipopolysaccharide layer as well as of capsular polysaccharides in Gram-negative bacteria. *N*-Acetyl-D-mannosamine (ManNAc) has been found to be, presumably, the strongest monosaccharidic ligand for the natural killer cells (NK-cells) activating protein NKR-P1 [1], and some ManNAc-containing oligosaccharides (e.g., Glc*p*NAc-β-(1→4)Man*p*NAc) have been identified to be strong immunoactivators [2]. Detection of ManNAc by the immune system is probably very important for recognizing bacterial infection, as this carbohydrate is an unambiguous signal of a microbial invader [3]. In the somatic cells (vertebrates) there are no structures composed of ManNAc. This carbohydrate is a precursor of sialic acid(s). The pathogenicity of some bacterial strains occurring in their R-forms (R stands for rough: virulent, containing ManNAc in the capsular structures) and S-forms (S stands for smooth: nonvirulent, lower level of ManNAc in the capsules) is related partly to the content of ManNAc. Thus, ManNAc units play a significant role in bacterial pathogenicity and virulence (e.g., *Streptococcus pneumoniae*) [4–5]. Surprisingly, glycosidases active upon β-Man*p*NAc and α-Man*p*NAc glycosides are not known so far. Therefore, the building blocks for the chemical synthesis of ManNAc-containing compounds, as well as the substrates for the hypothetical α-*N*-acetylmannopyranosidase, are required.

This paper describes new robust and effective methods for the synthesis of both *o*-nitrophenyl 2-acetamido-2-deoxy-α-D-mannopyranoside (**7**) and *p*-nitrophenyl 2-acetamido-2-deoxy-α-D-mannopyranoside (**8**) usable as practical chromogenic substrates for the screening of such enzymes.

## Results and Discussion

The first reported preparation of *p*-nitrophenyl 2-acetamido-2-deoxy-α-D-mannopyranoside (*p*NP-α-ManNAc, **8**) was described in [6], starting from 2-acetamido-2-deoxy-D-glucose and providing the product in 2% overall yield. We have previously published [7] a 9-step synthesis of *p*NP-α-ManNAc (**8**) from commercially available methyl α-D-glucopyranoside with an overall yield of 1.5%. Later, Popelová et al. [8] published a concise synthesis starting either from D-glucose (5 steps, 1.5% yield) or from methyl 4,6-*O*-benzylidene-α-D-glucopyranoside (5 steps, 23%). Unfortunately, in our hands this synthetic procedure did not afford the published yields, namely in the triflate and consequent azide substitution steps.

Oxazolines, such as **3**, have been used as glycosylation agents in the preparation of glycoproteins [9–10], various alcohol glycosides [11–12], phosphates [13] and oligosaccharides [12]. To the best of our knowledge, glycosylation of phenolic OH with oxazoline has not been accomplished so far. Therefore, we decided to test oxazoline **3** [14] glycosylation in our synthetic approach.

### Synthetic pathway

**Scheme 1:**
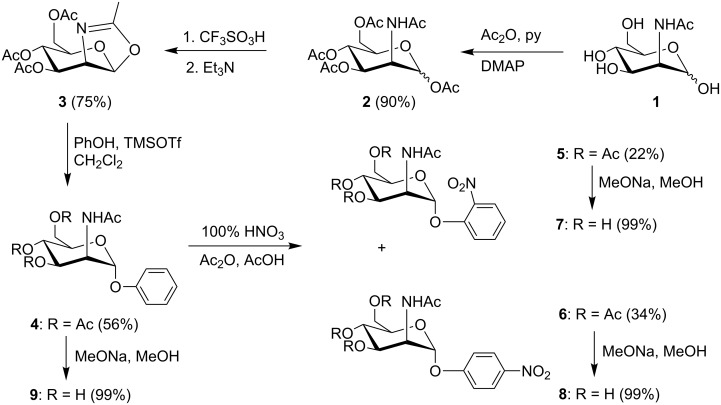
Synthetic pathway.

The synthesis (see Scheme 1) was started from commercially available 2-acetamido-2-deoxy-D-mannopyranose (**1**, ManNAc), which was converted to its peracetate **2** (Ac_2_O/py/DMAP (cat.), 90%). A crude mixture of peracetylated ManNAc **2** was treated with trifluoromethanesulfonic acid [14] affording oxazoline **3** in 75% yield.

The glycosylation of phenol and *p*-nitrophenol with oxazoline **3** was extensively tested. We tested a large array of reaction conditions, including variation of catalyst (copper(II) chloride [12,15], 2,2-diphenyl-1-picrylhydrazyl, zinc chloride, tin(IV) chloride) and solvent (CH_2_Cl_2_, THF, toluene, benzene), all of which were not successful. Finally, we found that trimethylsilyl trifluoromethanesulfonate in dichloromethane [11] was capable of catalyzing the oxazoline ring opening with phenol to afford α-phenyl glycoside **4** in a reasonable 56% yield. This reaction was also tested with *p*-nitrophenol; however, the required *p*-nitrophenyl glycoside **6** was produced under these conditions only in trace amounts (observed by TLC). Our and previously published results [11] indicate that the deactivation of phenol by the electron-withdrawing nitro group substantially decreases its reactivity in the glycosylation reaction with oxazoline and, on the other hand, the presence of electron-donating groups increases the yields.

The resulting phenyl glycoside **4** was treated with a solution of red fuming nitric acid in acetic acid [16], producing a mixture of *o*-nitrophenyl glycoside **5** (22% yield) and *p*-nitrophenyl glycoside **6** (34% yield) in approximately 2:3 ratio, which was separated by flash chromatography. Zemplén deacetylation of **4** and **5** afforded the title compounds **7** and **8** in almost quantitative yields.

## Conclusion

In conclusion, a simple and robust procedure for the synthesis of *o*- and *p*-nitrophenyl 2-acetamido-2-deoxy-α-D-mannopyranoside (**7** and **8**) from commercially available ManNAc, via its oxazoline, is described affording an overall total yield of both **7** and **8** of over 21%, including the purification steps.

## Experimental

### General methods

All chemicals were purchased from Sigma-Aldrich, except for 2-acetamido-2-deoxy-D-mannose, which was purchased from BIOSYNTH AG, Staad, CH. The reactions were monitored by TLC with precoated silica gel 60 F_254_ aluminium sheets from Merck, detected with UV light and/or charred with sulfuric acid (5% in EtOH). The compounds were purified either by column flash chromatography with silica gel 60 (230–240 mesh, Merck) or by gel permeation chromatography with Sephadex LH20 from Sigma Aldrich. The solvents were distilled and dried according to the standard procedures before use.

### NMR spectroscopy

NMR spectra were recorded on a Bruker Avance III 400 MHz spectrometer (400.13 MHz for ^1^H, 100.55 MHz for ^13^C at 30 °C) and a Bruker Avance III 600 MHz spectrometer (600.23 MHz for ^1^H, 150.93 MHz for ^13^C at 30 °C) in CD_3_OD or CDCl_3_ (Sigma-Aldrich). Residual signals of the deuterated solvents were used as internal standards (for CD_3_OD δ_H_ 3.330 ppm, δ_C_ 49.30 ppm, for CDCl_3_ δ_H_ 7.265 ppm, δ_C_ 77.23 ppm). NMR experiments ^1^H NMR, ^13^C NMR, gCOSY, gHSQC, and gHMBC were performed using the manufacturer’s software. ^1^H NMR and ^13^C NMR spectra were zero filled to fourfold data points and multiplied by a window function before Fourier transformation. A two-parameter double-exponential Lorentz–Gauss function was applied for ^1^H to improve resolution, and line broadening (1 Hz) was applied to get a better ^13^C signal-to-noise ratio. Chemical shifts are given on a δ-scale with the digital resolution justifying the reported values to three (δ_H_) or two (δ_C_) decimal places.

The anomeric configuration of *manno-*structures was determined based on the value of ^1^*J* (C-1, H-1) [17], which was measured by using gHMQC or gHSQC with retained direct coupling constants between directly bonded carbon and hydrogen.

### Mass spectrometry

Mass spectra were measured on MALDI–TOF/TOF ultraFLEX III mass spectrometers (Bruker-Daltonics). Positive spectra were calibrated externally by using the monoisotopic [M + H]^+^ ions of PepMixII calibrant (Bruker-Daltonics). For the MALDI experiment 0.4 μL of sample dissolved in 50% acetonitrile was allowed to dry at ambient temperature on the target and overlaid with matrix solution (either 2,5-dihydroxybenzoic acid, DHB or α-cyano-4-hydroxycinnamic acid, CCA). The spectra were collected in reflectron mode.

**Phenyl 2-acetamido-2-deoxy-3,4,6-tri-*O*-acetyl-α-D-mannopyranoside (4):** 2-Methyl-(3,4,6-tri-*O*-acetyl-1,2-dideoxy-β-D-mannopyrano)-[2,1:4’,5’]-2-oxazoline [18] **3** (280 mg, 0.85 mmol) was dissolved in 10 mL of dry dichloromethane. To this solution, phenol (250 mg, 2.66 mmol, 3 equiv) and trimethylsilyl trifluoromethanesulfonate (0.2 mL, 0.73 mmol, 1 equiv) were added. The reaction mixture was stirred at room temperature for 3 days (monitored by TLC on silica gel, chloroform/acetone 8:1). The reaction mixture was evaporated to dryness in vacuo. The product was isolated by column chromatography (silica gel, chloroform/acetone 20:1) as a white solid (200 mg, 56%). ^1^H NMR (400 MHz, CDCl_3_) δ 2.015 (s, 3H, 6-Ac), 2.022 (s, 3H, 3-Ac), 2.048 (s, 3H, 4-Ac), 2.080 (s, 3H, 2-Ac), 4.022 (dd, *J* = 2.4, 12.2 Hz, 1H, H-6u), 4.104 (ddd, *J* = 2.4, 5.6, 10.2 Hz, 1H, H-5), 4.265 (dd, *J* = 5.6, 12.2 Hz, 1H, H-6d), 4.821 (ddd, *J* = 1.6, 4.7, 8.9 Hz, 1H, H-2), 5.168 (dd, *J* = 10.2, 10.2 Hz, 1H, H-4), 5.475 (d, *J* = 1.6 Hz, 1H, H-1), 5.564 (dd, *J* = 4.7, 10.2 Hz, 1H, H-3), 5.919 (d, *J* = 8.9 Hz, 1H, 2-NH), 7.047 (m, 1H, H-*para*), 7.075 (m, 2H, H-*ortho*), 7.291 (m, 2H, H-*meta*); ^13^C NMR (100 MHz, CDCl_3_) δ 20.60, 20.70 (3 x q, 3-Ac, 4-Ac, 6-Ac), 23.29 (q, 2-Ac), 50.51 (d, C-2), 62.14 (t, C-6), 65.99 (d, C-4), 68.67 (d, C-5), 68.89 (d, C-3), 97.12 (d, C-1), 116.49 (d, C-*ortho*), 122.99 (d, C-*para*), 129.56 (d, C-*meta*), 155.60 (s, C-*ipso*), 169.82 (s, 3-CO), 169.94 (s, 4-CO), 170.15 (s, 2-CO), 170.40 (s, 6-CO).

***o*-Nitrophenyl 2-acetamido-2-deoxy-3,4,6-tri-*O*-acetyl-α-D-mannopyranoside (5) and *p*-nitrophenyl 2-acetamido-2-deoxy-3,4,6-tri-*O*-acetyl-α-D-mannopyranoside (6):** Compound **4** (1.1 g, 2.59 mmol) was dissolved in a mixture of acetic anhydride (10 mL) and glacial acetic acid (2 mL). The solution was cooled to 0 °C in an ice bath, and red fuming nitric acid (≥99.5%, 0.5 mL) was added in one portion under stirring. The reaction mixture was stirred overnight, allowing the ice bath to melt and warm up to ambient temperature. The reaction mixture was diluted by an ice–water mixture (50 mL), stirred for an additional 30 min, and neutralized by the saturated aqueous solution of NaHCO_3_. After extraction with methylene dichloride (3 × 50 mL), the combined organic layers were washed with 50 mL of sodium bicarbonate solution and 50 mL of water. After drying (Na_2_SO_4_) the organic phase was evaporated and chromatographed (silica gel, chloroform/acetone 5:1). *o*-Nitrophenyl derivative **5** was isolated as a white solid (270 mg, 22%) and *p*-nitrophenyl derivative **6** was isolated as a white solid (410 mg, 34%).

(**5**) ^1^H NMR (400 MHz, CDCl_3_) δ 2.032 (s, 3H, 3-Ac), 2.039 (s, 3H, 6-Ac), 2.080 (s, 3H, 4-Ac), 2.104 (s, 3H, 2-Ac), 4.042 (dd, *J* = 2.2, 12.3 Hz, 1H, H-6u), 4.212 (ddd, *J* = 2.2, 5.2, 10.2 Hz, 1H, H-5), 4.286 (dd, *J* = 5.2, 12.3 Hz, 1H, H-6d), 4.812 (ddd, *J* = 1.8, 4.8, 7,7 Hz, 1H, H-2), 5.213 (dd, *J* = 10.2, 10.2 Hz, 1H, H-4), 5.593 (dd, *J* = 4.8, 10.2 Hz, 1H, H-3), 5.750 (d, *J* = 1.8 Hz, 1H, H-1), 5.909 (d, *J* = 7.7 Hz, 1H, 2-NH), 7.170 (ddd, *J* = 1.2, 7.4, 8.2 Hz, 1H, H-4´), 7.329 (dd, *J* = 1.2, 8.5 Hz, 1H, H-6´), 7.544 (ddd, *J* = 1.7, 7.4, 8.5 Hz, 1H, H-5´), 7.905 (dd, *J* = 1.7, 8.2 Hz, 1H, H-3´); ^13^C NMR (100 MHz, CDCl_3_) δ 20.62, 20.63, 20.65 (3 x q, 3-Ac, 4-Ac, 6-Ac), 23.28 (q, 2-Ac), 50.68 (d, C-2), 61.97 (t, C-6), 65.52 (d, C-4), 68.33 (d, C-3), 69.68 (d, C-5), 97.34 (d, C-1), 117.24 (d, C-6´), 122.82 (d, C-4´), 125.88 (d, C-3´), 134.09 (d, C-5´), 140.58 (s, C-2´), 148.66 (s, C-1´), 169.20 (s, 3-CO), 170.07 (s, 4-CO), 170.32 (s, 6-CO), 170.47 (s, 2-CO).

(**6**) ^1^H NMR (400 MHz, CDCl_3_) δ 2.028 (s, 3H, 6-Ac), 2.047 (s, 3H, 3-Ac), 2.065 (s, 3H, 4-Ac), 2.109 (s, 3H, 2-Ac), 4.02* (m, 1H, H-5), 4.03* (m, 1H, H-6u), 4.273 (dd, *J* = 5.6, 12.4 Hz, 1H, H-6d), 4.818 (ddd, *J* = 1.6, 4.8, 8.4 Hz, 1H, H-2), 5.196 (dd, *J* = 10.2, 10.3 Hz, 1H, H-4), 5.555 (dd, *J* = 4.8, 10.3 Hz, 1H, H-3), 5.629 (d, *J* = 1.6 Hz, 1H, H-1), 5.905 (d, *J* = 8.4 Hz, 1H, 2-NH), 7.205 (m, 2H, H-*ortho*), 8.225 (m, 2H, H-*meta*) (* = HSQC readout); ^13^C NMR (100 MHz, CDCl_3_) δ 20.59, 20.62, 20.67 (3 x q, 3-Ac, 4-Ac, 6-Ac), 23.28 (q, 2-Ac), 50.42 (d, C-2), 61.94 (t, C-6), 65.58 (d, C-4), 68.35 (d, C-3), 69.31 (d, C-5), 96.89 (d, C-1), 116.47 (d, C-*ortho*), 125.82 (d, C-*meta*), 143.19 (s, C-*para*), 160.22 (s, C-*ipso*), 169.68 (s, 3-CO), 169.86 (s, 4-CO), 170.26 (s, 6-CO), 170.36 (s, 2-CO).

***o*-Nitrophenyl 2-acetamido-2-deoxy-α-D-mannopyranoside (7):** Compound **5** (270 mg, 0.58 mmol) was dissolved in dry methanol (2 mL) and three drops of NaOMe in MeOH (35%, w/w) were added. The reaction mixture was stirred at ambient temperature for 20 minutes. The solution was directly loaded onto the gel permeation chromatography column (Sephadex LH-20) with methanol as a mobile phase (2 mL/min, UV detection 254 nm). The product was isolated as a white solid (197 mg, 99%). ^1^H NMR (400 MHz, CD_3_OD) δ 2.070 (s, 3H, 2-Ac), 3.660 (ddd, *J* = 2.3, 4.5, 10.0 Hz, 1H, H-5), 3.761 (dd, *J* = 2.5, 12.0 Hz, 1H, H-6u), 3.756 (dd, *J* = 9.7, 9.9 Hz, 1H, H-4), 3.824 (dd, *J* = 4.5, 12.0 Hz, 1H, H-6d), 4.183 (dd, *J* = 4.9, 9.7 Hz, 1H, H-3), 4.541 (dd, *J* = 1.7, 4.9 Hz, 1H, H-2), 5.689 (d, *J* = 1.7 Hz, 1H, H-1), 7.188 (ddd, *J* = 1.2, 7.4, 8.1 Hz, 1H, H-4´), 7.515 (dd, *J* = 1.2, 8.5 Hz, 1H, H-6´), 7.612 (ddd, *J* = 1.7, 7.4, 8.5 Hz, 1H, H-5´), 7.854 (dd, *J* = 1.7, 8.1 Hz, 1H, H-3´); ^13^C NMR (100 MHz, CD_3_OD) δ 22.93 (q, 2-Ac), 54.36 (d, C-2), 62.23 (t, C-6), 68.14 (d, C-4), 70.43 (d, C-3), 76.04 (d, C-5), 99.65 (d, C-1), 118.74 (d, C-6´), 123.58 (d, C-4´), 126.51 (d, C-3´), 135.52 (d, C-5´), 142.17 (s, C-2´), 150.50 (s, C-1´), 174.61 (s, 2-CO); MS–MALDI–TOF (*m*/*z*): 365.1 [M + Na]^+^ (DHB), 365.1 [M + Na]^+^ (CCA).

***p*-Nitrophenyl 2-acetamido-2-deoxy-α-D-mannopyranoside (8):** Compound **6** (400 mg, 0.85 mmol) was deacetylated and purified as described for compound **7** yielding **8** as a white solid (290 mg, 99%). ^1^H NMR (400 MHz, CD_3_OD) δ 2.078 (s, 2-Ac3H, ), 3.562 (ddd, *J* = 2.4, 4.7, 9.9 Hz, 1H, H-5), 3.746 (dd, *J* = 2.4, 12.0 Hz, 1H, H-6u), 3.752 (dd, *J* = 9.7, 9.9 Hz, 1H, H-4), 3.812 (dd, *J* = 4.7, 12.0 Hz, 1H, H-6d), 4.164 (dd, *J* = 4.9, 9.7 Hz, 1H, H-3), 4.544 (dd, *J* = 1.7, 4.9 Hz, 1H, H-2), 5.657 (d, *J* = 1.7 Hz, 1H, H-1), 7.300 (m, 2H, H-*ortho*), 8.230 (m, 2H, H-*meta*). ^13^C NMR (100 MHz, CD_3_OD) δ 22.93 (q, 2-Ac), 54.28 (d, C-2), 62.23 (t, C-6), 68.20 (d, C-4), 70.51 (d, C-3), 75.79 (d, C-5), 99.09 (d, C-1), 118.05 (d, C-*ortho*), 127.00 (d, C-*meta*), 144.21 (s, C-*para*), 162.80 (s, C-*ipso*), 174.66 (s, 2-CO); MS–MALDI–TOF (*m*/*z*): 365.1 [M + Na]^+^ (DHB), 365.1 [M + Na]^+^ (CCA).

**Phenyl 2-acetamido-2-deoxy-α-D-mannopyranoside (9):** Compound **4** (50 mg, 0.12 mmol) was deacetylated and purified as described for compound **7**, yielding **9** as a white solid (35 mg, 99%). ^1^H NMR (400 MHz, CD_3_OD) δ 2.067 (s, 3H, 2-Ac), 3.654 (m, 1H, H-5), 3.73* (m, 1H, H-6u), 3.75* (m, 1H, H-4), 3.838 (dd, *J* = 4.2, 11.9 Hz, 1H, H-6d), 4.178 (dd, *J* = 4.8, 9.6 Hz, 1H, H-3), 4.525 (dd, *J* = 1.7, 4.8 Hz, 1H, H-2), 5.466 (d, *J* = 1.7 Hz, 1H, H-1), 7.023 (m, 1H, H-*para*), 7.110 (m, 2H, H-*ortho*), 7.298 (m, 2H, H-*meta*) (* - HSQC readouts); ^13^C NMR (100 MHz, CD_3_OD) δ 22.95 (q, 2-Ac), 54.64 (d, C-2), 62.22 (t, C-6), 68.35 (d, C-4), 70.81 (d, C-3), 75.06 (d, C-5), 99.22 (d, C-1), 118.00 (d, C-*ortho*), 123.79 (d, C-*para*), 130.83 (d, C-*meta*), 157.95 (s, C-*ipso*), 174.53 (s, 2-CO); MS–MALDI–TOF (*m*/*z*): 320.1 [M + Na]^+^ (DHB), 320.1 [M + Na]^+^ (CCA).

## References

[1] Krist P, Herkommerová-Rajnochová E, Rauvolfová J, Semeňuk T, Vavrušková P, Pavlíček J, Bezouška K, Petruš L, Křen V (2001). Biochem Biophys Res Commun.

[2] Sedmera P, Přikrylová V, Bezouška K, Rajnochová E, Thiem J, Křen V (1998). J Carbohydr Chem.

[3] Attolino E, Bonaccorsi F, Catelani G, D'Andrea F, Křenek K, Bezouška K, Křen V (2008). J Carbohydr Chem.

[4] Lee C J, Fraser B A (1980). J Biol Chem.

[5] Lee C J, Banks S D, Li J P (1991). Crit Rev Microbiol.

[6] Yoshimura J, Sakai H, Oda N, Hashimoto H (1972). Bull Chem Soc Jpn.

[7] Krist P, Kuzma M, Pelyvás I F, Simerská P, Křen V (2003). Collect Czech Chem Commun.

[8] Popelová A, Kefurt K, Hlaváčková M, Moravcová J (2005). Carbohydr Res.

[9] Rich J R, Withers S G (2009). Nat Chem Biol.

[10] Hollósi M, Kollát E, Laczkó I, Medzihradsky K F, Thurin J, Otvös L (1991). Tetrahedron Lett.

[11] Lopin C, Jacquinet J-C (2006). Angew Chem, Int Ed.

[12] Wittmann V, Lennartz D (2002). Eur J Org Chem.

[13] Heidlas J E, Lees W J, Pale P, Whitesides G M (1992). J Org Chem.

[14] Freese S J, Vann W F (1996). Carbohydr Res.

[15] Subramanian V, Moume-Pymbock M, Hu T, Crich D (2011). J Org Chem.

[16] Weissmann B (1966). J Org Chem.

[17] Bock K, Pedersen C (1974). J Chem Soc, Perkin Trans 2.

[18] Yamayaki T, Warden C H, Herscovics A, Jeanloz R W (1980). Carbohydr Res.

